# The Marriage of Syphilitics

**Published:** 1919-07-12

**Authors:** 


### THE EDITOR'S LETTER-BOX.

### The Marriage of Syphilitics.

*To the Editor of* THE HOSPITAL.

IN vain is a net spread in the sight of any bird. Dr. Hawthorne invites us to engage in a discussion on the precise meaning of venereal disease, a discussion that might easily be prolonged for months, and that is quite unnecessary, since everyone has a notion of it quite precise enough for practical purposes such as that of this discussion. Surely Dr. Hawthorne must recognise the difference, with reference to marriage, between venereal diseases and the other diseases he mentions. Insanity and epilepsy are not contagious. Tubercle is rarely communicated from one spouse to another. Venereal diseases are certain to be communicated. The difference is quite sufficient to warrant and necessitate different treatment.

Dr. Hawthorne's remedy of the certificate of health would be nugatory unless the production of such a certificate were made obligatory by statute, and of this there is not the least probability; but if it were made obligatory it would incontestably involve a violation of professional secrecy. From this violation there is no escape if the marriage of sufferers from venereal disease is to be prevented.

An analogous case may be found in the sister profession of law, in which the practitioner is legally protected from being required to violate professional confidence. He may not reveal the confession of a client that the client has committed a crime, but he is bound to reveal the fact that his client intends to commit a crime. To communicate venereal disease to another person is now, in certain circumstances, a crime, and a syphilitic who proposes to marry, knowing that he suffers from syphilis, proposes to commit this crime, and ought to be prevented from committing it, professional secrecy notwithstanding.

The last objection of Dr. Hawthorne is directed against a position that we do not occupy. It is very unlikely that the marriage of syphilitics will ever be wholly prevented, but that does not seem to us a valid reason for refusing to prevent it when we can. Even smallpox is not entirely prevented in this country, nor is yellow fever entirely abolished in Panama; but if Dr. Hawthorne considers that the enormous diminution of both has not been worth while, since cases of each do still occasionally occur, we fancy he will be alone in his opinion. Even if a certificate of health were made a compulsory preliminary to marriage, it would not prevent the occasional marriage of a syphilitic, any more than the certificate of the doctor of a life assurance company saves the company from ever incurring a loss. In this imperfect world we have to be content with doing the best we can with the means at our disposal, and if we never act until we can be sure that our action will be successful in every case, we shall never act at all.

THE WRITER OF THE ARTICLE.

### Physiognomy.

*To the Editor of* THE HOSPITAL.

